# Paediatric temporal bone fractures: a single centre experience

**DOI:** 10.1017/S0022215124001397

**Published:** 2025-02

**Authors:** Arash Algouneh, Edward Madou, Karan Gandhi, Josee Paradis, M. Elise Graham, Julie Strychowsky, Murad Husein, Peng You

**Affiliations:** 1 Schulich School of Medicine & Dentistry, Western University, London, Ontario, Canada; 2 Department of Otolaryngology – Head and Neck Surgery, London Health Sciences Centre, London, Ontario, Canada

**Keywords:** hearing loss, sensorineural hearing loss, facial nerve, vertigo, Inner ear, computerised tomography

## Abstract

**Objective:**

This study aimed to evaluate clinical characteristics, treatments and outcomes of paediatric temporal bone fractures at our institute.

**Methods:**

A retrospective study of paediatric skull fractures confirmed by imaging from January 2010 to December 2022. Data on demographics, clinical presentations, injury mechanisms and complications were analysed, and fractures were categorised into otic capsule sparing (OCS) and violating (OCV).

**Results:**

Of 369 skull fracture cases, 88 (24 per cent) involved temporal bones, predominantly caused by falls and vehicle accidents. Common symptoms were loss of consciousness, hematoma, and hemotympanum, with complications like facial nerve injury and cerebrospinal fluid leaks in 3.4 per cent of cases. OCV fractures led to more severe complications, including hearing loss. Audiology showed 65 per cent without hearing impairment, while others had various degrees of loss.

**Conclusion:**

Paediatric temporal bone fractures, particularly OCV types, pose significant challenges. Early detection and thorough management are vital, underscoring the need for consistent data collection and regular audiometric monitoring.

## Introduction

Our study aims to review the clinical characteristics, therapeutic approaches and subsequent outcomes of paediatric temporal bone fractures. With a primary objective of determining these fractures' prevalence and associated complications, this research additionally seeks to guide early intervention strategies rooted in data from a tertiary care hospital network.

The temporal bone is a complex structure housing critical elements such as the middle ear, inner ear, and facial nerve.^1^ Temporal bone fractures can lead to complications like conductive and sensorineural hearing loss, facial nerve injury/palsy, cerebrospinal fluid leaks and other neurological issues.^1^ These complications can significantly impact paediatric patients, affecting developmental milestones, language acquisition, cognitive growth and social interactions.^2,3^

Currently, management strategies for temporal bone injuries are primarily based on adult data, underscoring the need for paediatric-specific research. This study aims to fill that gap by providing insights into paediatric temporal bone fractures' unique challenges and outcomes.

## Materials and methods

A retrospective cohort study was conducted on patients aged 17 years or younger who presented to London Health Sciences Centre (LHSC), a tertiary care hospital network, from January 2010 to December 2022 with a skull fracture. Ethical approval was granted by the research ethics committee at the Lawson Health Research Institute of London Health Sciences Centre (Study number ReDA 12828). Charts were reviewed to identify specific cases with temporal bone fractures. The inclusion criterion was a head computed tomography (CT) at the time of injury confirming the fracture. Cases were excluded if they had incomplete clinical or imaging data necessary for the analysis.

Data collection included demographics, clinical presentation, mechanism of injury and complications. Information pertaining to initial Otolaryngology - Head and Neck Surgery (OHNS) consultations, and subsequent follow-ups were recorded. Temporal bone fractures were classified using the otic capsule sparing (OCS) versus otic capsule violating (OCV) system, diagnosed by board-certified radiologists.^4^ Clinical documentation provided details on signs and symptoms, capturing elements like hemotympanum, otorrhea, Battle sign (mastoid ecchymosis), periorbital ecchymosis and others. Additionally, charts review noted any instances of hearing loss, facial nerve injury and intracranial injuries. Data regarding associated skull fractures were also compiled. Post-injury audiometric assessments utilised pure-tone audiometry (PTA). Calculation was done in accordance with standardised format by using threshold at 0.5-, 1-, 2-, and 3-kHz rounded to the nearest whole number. When the threshold at 3 kHz was missing, the average of the thresholds at 2 and 4 kHz was used.^5^ When necessary, hearing loss outcomes were categorised as conductive (CHL), sensorineural (SNHL), or mixed, and are reported in decibel hearing level (dB).

Statistical analyses were carried out with GraphPad Prism 8. Statistical significance was calculated using multiple Student's *t*-test. The level of statistical significance is indicated in the figures.

## Results and analysis

Between 2010 and 2022, 369 patients were assessed with a skull fracture. Of these, 91 (25 per cent) were diagnosed with a temporal bone fracture. Three cases were excluded from analysis due to lack of relevant clinical data. The average age of this cohort of 88 patients was 8.6 years, with a male-to-female ratio of 1.3:1. Of the total, 42 patients (48 per cent) underwent an initial OHNS consultation, with 33 (38 per cent) returning for follow-up. On average, 1.47 follow-up encounters occurred after the initial follow-up. Patient demographics are presented in Table 1.

**Table 1.** Patient demographics

Age (Years)		
Mean Age	8.56	
Median Age	(5.44)	

	Patients (n)	%
Male/female	52/36	59%/41%
		
	Patients (n)	%/Median
Otolaryngology consultation	42	48%
Otolaryngology follow-up	33	38%
No. of additional follow-ups	1.47	1.80

**Figure 1.**
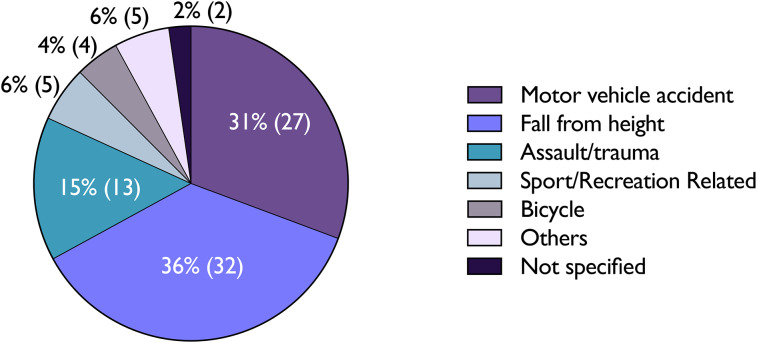
Mechanisms of Injury of Pediatric Temporal Bone Fracture

**Figure 2.**
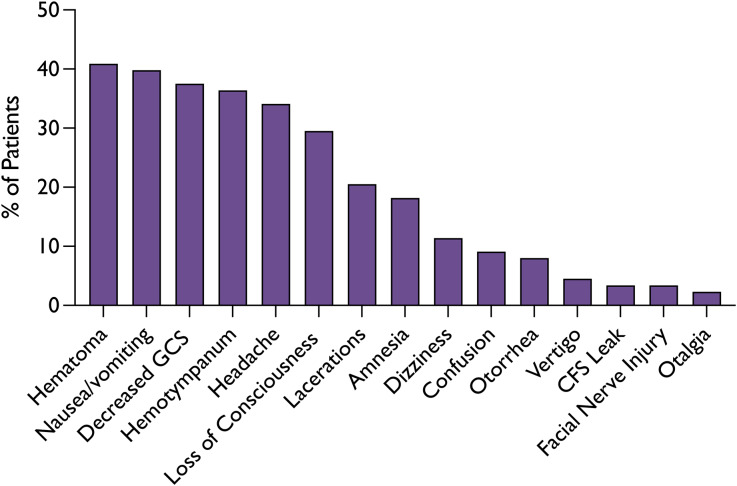
Clinical Presentations of Pediatric Temporal Bone Fracture

Falls from height were most common mechanism of injury, accounting for 36.4 per cent of cases (32 patients), closely followed by motor vehicle accidents (MVAs) at 30.7 (27 patients) (Figure 1). The clinical presentations commonly observed were decreased or loss of consciousness, hematoma, hemotympanum, headaches, nausea and vomiting (Figure 2). Notably, 24 patients (27 per cent) documented subjective hearing loss close to the time of the accident. Other classical physical indicators of basilar skull fractures, such as cerebrospinal fluid (CSF) rhinorrhoea (3.4 per cent) and Battle sign (0), were relatively rare.

**Figure 3.**
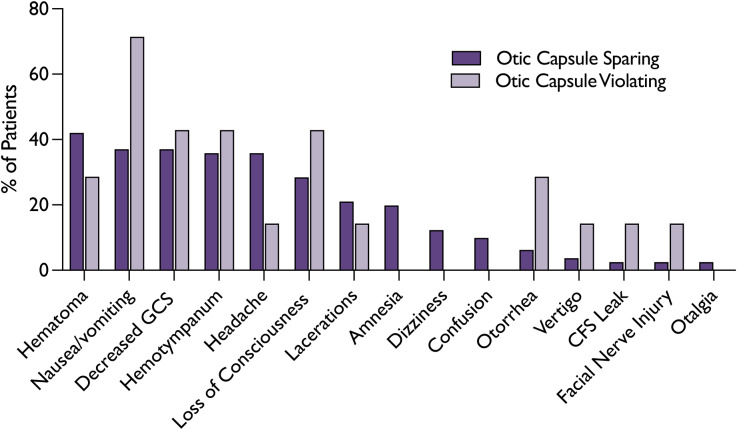
Clinical Presentations Based on Temporal Bone Fracture Type

Of the 81 patients with unilateral temporal bone fractures, 54 per cent (44/81) were on the right side. The remaining seven patients had bilateral temporal bone fractures. Concomitant fractures in other regions were observed in 54 per cent of the patients (Table 2). Of note, eight patients (9 per cent) were diagnosed with OCV fractures through CT scans. A comparative evaluation revealed that patients with OCV fractures experienced more complications. These include otorrhea (28.6 vs. 6.2 per cent), vertigo (14.3 vs. 3.7 per cent), CSF leaks (14.3 vs. 2.5 per cent) and facial nerve injuries (14.3 vs. 2.5 per cent) than their OCS fracture counterparts (Figure 3). While a comparative evaluation indicated higher incidences of complications, the study was not sufficiently powered to detect statistical significance for these differences.

**Table 2.** Fracture characteristics

Laterality of Fracture	Patients (N)	%
Right	44	50
Left	37	42
Bilateral	7	8
		
	Patients (n)	%
Other associated skull fractures	48	54%

Focusing on functional neural impairment (FNI) and CSF leaks, three patients (3.4 FNI) experienced FNI, and another three patients (3.4 FNI) had a CSF leak, with one of these patients presenting with both FNI and CSF leak (Figure 2). The first patient, with injuries arising from an MVA, had bilateral OCV fractures, resulting in left-sided SNHL, and right-sided mixed hearing loss with ossicular discontinuity. This patient also developed a left-sided FNI, which resolved spontaneously. The second patient, following an OCS fracture, also exhibited a transient FNI. The third patient sustained an OCV fracture, potentially linked to a CSF leak. While the leak spontaneously resolved, persistent right-sided FNP and SNHL remained post-accident. None of the patients received operative interventions. Data for electroneurography was not available. Regarding CSF leaks, one patient with an OCS fracture from an MVA had a CSF leak which was conservatively managed, leading to spontaneous resolution. Another patient, having suffered an OCV fracture from a fall, was treated initially with acetazolamide at a dose of 25mg/kg, which was titrated up to 75mg/kg q.i.d. (four times a day), successfully resolving the CSF leak.

**Figure 4.**
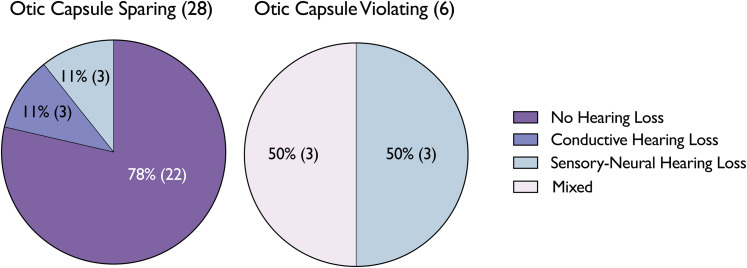
Audiology Assessments Categorized by Temporal Bone Fracture Type.

**Figure 5.**
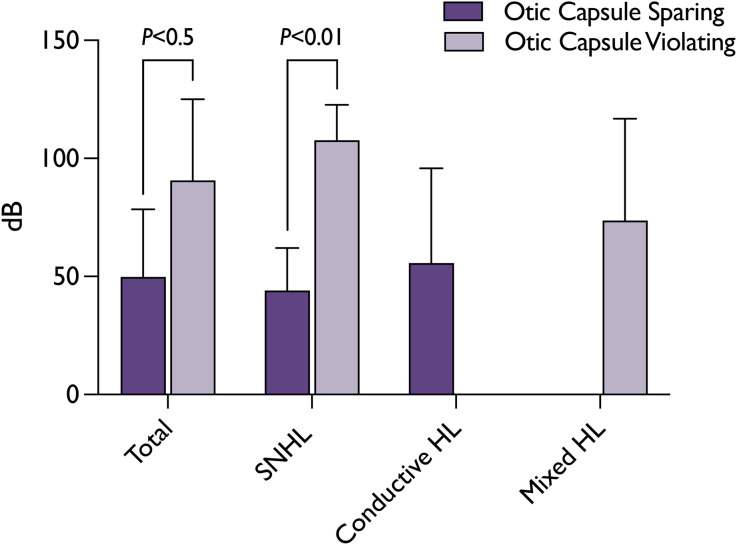
Post-injury audiometric puretone average (PTA) of effected ear. Mean ± standard deviation is shown. Y-axis provides PTAs in dB hearing level. Statistical significance was calculated using multiple Student’s t-test.

Of the 34 patients (39 FNI) who underwent an audiological assessment, 7 (21 FNI) were evaluated while in-patient, with the remainder receiving their assessments around the time of their first follow-up appointment. The findings were as follows: 22 patients (65 per cent) exhibited no hearing impairment; 3 patients (9 per cent) presented with CHL (PTA 55.67dB, SD 32.79); 6 (18 per cent) had SNHL (PTA 75.83dB, SD 34.60); and the remaining 3 (9 per cent) displayed mixed hearing loss (PTA 73.67dB, SD 35.24). Grouping the results by fracture type, patients with OCV fractures exhibited a significantly higher incidence of hearing loss with significantly higher PTAs (100 per cent vs. 21.4 per cent, PTA 90.67dB vs. 49.83dB, *p* = 0.0492). Furthermore, those with OCV fractures suffered either from SNHL (three patients, 50 per cent, PTA 107.67dB, SD 12.26) and mixed hearing loss (three patients, 50 per cent, PTA 73.67dB, SD 35.24). These results are demonstrated in Figure 4 and 5. Of note, two patients with profound SNHL were evaluated for cochlear implant candidacy.

## Discussion

Temporal bone fractures in paediatric patients present significant clinical challenges due to their potential complications which may be long-lasting or permanent. Our retrospective study, conducted at LHSC between 2010 and 2022, complements the existing literature, providing vital data on fracture prevalence, manifestations and outcomes.

There is a distinct variation in the mechanisms of injury between paediatric and adult patients. Specifically, falls from height (36.4 per cent) and MVAs (30.7 per cent) were the leading causes of temporal bone fractures in children, in contrast to adult populations, where MVAs are predominantly responsible for temporal bone fractures.^6,7^ This pattern aligns with existing paediatric literature, which consistently cites falls as the most common mechanism of injury in paediatric populations.^8,9^ The variance in causative factors can be attributed to developmental and behavioural differences between children and adults and varying exposure to risk factors. The diverse range of injury mechanisms, including MVAs, falls, biking, and other recreational activities, underscores the complex and multifaceted nature of paediatric trauma. Understanding the pattern of injury may help direct preventative strategies. Such strategies should be focused on environments where these accidents are more likely to occur and should be age-specific to address the changing risk profiles as children grow. By understanding and addressing the unique patterns of injury in the paediatric population, we can better develop effective interventions to reduce the incidence and severity of temporal bone fractures in children.

The incidence of OCV fractures in our paediatric cohort was 9 per cent, mirroring the rates reported in adult populations, indicating that the propensity for such fractures transcends age groups.^10,11^ The prognostic implications are profound, as OCV fractures adversely impact inner ear structures due to the substantial force of injury, leading to complications such as SNHL, otorrhea and vertigo.^12^ Facial nerve injury incidence in our study was 3.4 per cent, aligning with the reported literature range of 3–7 per cent.^13^ Within our cohort, only one patient had permanent nerve paralysis and the other two had transient facial nerve dysfunction. Despite their relative rarity, the significant morbidities associated with OCV fractures necessitate careful consideration in the clinical management of temporal bone fractures. The severity of potential complications demands that clinicians remain vigilant, ensuring thorough monitoring and intervention to lessen the impact on affected paediatric patients.

Hearing loss was a common consequence of paediatric temporal bone fractures in our study, corroborating the findings of Thorén *et al.* who noted frequent otologic symptoms in such cases.^14^ Our data highlight the critical need for prompt audiometric assessment post-fracture to identify and manage hearing impairment early. Implementing such assessments ensures that any degree of hearing loss can be promptly identified and managed. In profound SNHL, early detection is critical, as evidenced by our identification of patients potentially suitable for cochlear implantation, with PTA in the profound range, greater than 90 dB. Earlier cochlear implantation has been demonstrated to improve outcomes in cochlear implant recipients and reduces the risk of labyrinthitis ossificans which can rarely result from temporal bone trauma. Therefore, early audiometric testing should be an integral part of the management protocol for patients with temporal bone fractures to ensure optimal care and outcomes. It is a crucial step in the immediate and long-term management of paediatric patients with temporal bone fractures, providing a pathway to quantify the extent of hearing loss and facilitate timely rehabilitative strategies.

Notably, our findings revealed that three patients with an OCS fracture experienced persistent mid-high frequency SNHL for over a year post-injury. Treating clinicians in the cases ascribed this to a probable cochlear concussion, a descriptor for trauma to the membranous labyrinth in the absence of a discernible fracture. The theories suggesting mechanisms of hearing loss, such as membranous cochlea rupture from CSF pressure waves, disruption of cochlear microcirculation or cochlear haemorrhage, provide a framework to understand the underlying pathophysiology in our findings.^15^ Specifically, our data may support the hypothesis that, even in the absence of a fracture, significant inner ear damage can manifest as long-term auditory impairment. This correlation underscores the necessity for further investigation into the microvascular and intracochlear pathology that may contribute to persistent SNHL following head trauma and validates the need for heightened clinical awareness of such auditory sequelae in patients with OCS fractures.

With regards to limitations, the retrospective nature of our study inherently constrained our ability to apply standardised reporting, culminating in gaps within our dataset, particularly in audiometric evaluations. Only 36 per cent of our patients underwent formal hearing assessments, a deficiency mirrored in the field: Thorén *et al.* observed that a mere 24.7 per cent of their cohort received otolaryngology consultations, Waissbluth *et al*. reported 15 per cent of patients bypassed hearing tests, and Wexler *et al*. found audiometry was completed in only 28.3 per cent (13 out of 46) of cases.^9,14,16^ The data were collected from a tertiary centre. It is possible patients received further evaluations elsewhere. Alternatively, concomitant critical injuries could have diverted from comprehensive follow-ups. This underlines the need for standardised evaluation protocols for paediatric patients with temporal bone fractures, encompassing routine hearing screenings and prompt otolaryngology referrals. Our study's limited data also restricted a thorough investigation into the prevalence of cochlear concussion, a notable point of concern, given that two patients with profound SNHL were evaluated for cochlear implant candidacy. Regular audiometric follow-ups are crucial for timely diagnosis and intervention and vital for ensuring the best patient outcomes. Hence, while providing valuable insights, our study accentuates the need for systematic assessment protocols to ensure comprehensive care and precise data collection in this vulnerable population.

Our study underscores the importance of early detection and comprehensive care in managing temporal bone fractures in children. As research in this area progresses, prospective and broader cohort evaluations can offer more in-depth insights, enhancing paediatric healthcare approaches.

## Conclusion

Our study sheds light on the clinical presentation, therapeutic approaches and outcomes of paediatric temporal bone fractures, aiming to determine their prevalence and associated complications and to guide early intervention strategies. Despite the inherent limitations of a retrospective study, our findings provide valuable paediatric-specific data to the predominantly adult-focused literature on temporal bone fractures.

A significant finding was the lack of audiological assessment in approximately two-thirds of the children, which can be attributed to several factors. The presence of concomitant critical injuries often diverted attention from comprehensive follow-ups, and variations in clinical practice and resource availability may have also contributed. This highlights the need for standardised protocols to ensure that all patients receive appropriate and timely audiological evaluations.

We found that, while falls were the most common cause of fractures in children, differing from adults, the clinical patterns and complications, such as hearing loss and facial nerve injuries, had significant developmental impacts. Notably, OCV fractures resulted in more severe complications.

Our research emphasises the need for early and systematic audiometric assessments, vigilant monitoring for complications, and tailored intervention strategies to mitigate the long-term consequences of these injuries. Future studies with standardised protocols and prospective design are essential to build upon our findings, enhance patient care, and guide preventative measures for paediatric temporal bone fractures.
